# Protease-Activated Receptor-2 Deficiency Attenuates Atherosclerotic Lesion Progression and Instability in Apolipoprotein E-Deficient Mice

**DOI:** 10.3389/fphar.2017.00647

**Published:** 2017-09-14

**Authors:** Pengfei Zuo, Zhi Zuo, Yueyue Zheng, Xin Wang, Qianxing Zhou, Long Chen, Genshan Ma

**Affiliations:** Department of Cardiology, Zhongda Hospital Affiliated to Southeast University Nanjing, China

**Keywords:** atherosclerosis, plaque stability, protease-activated receptor-2, inflammation, macrophage

## Abstract

Inflammatory mechanisms are involved in the process of atherosclerotic plaque formation and rupture. Accumulating evidence suggests that protease-activated receptor (PAR)-2 contributes to the pathophysiology of chronic inflammation on the vasculature. To directly examine the role of PAR-2 in atherosclerosis, we generated apolipoprotein E/PAR-2 double-deficient mice. Mice were fed with high-fat diet for 12 weeks starting at ages of 6 weeks. PAR-2 deficiency attenuated atherosclerotic lesion progression with reduced total lesion area, reduced percentage of stenosis and reduced total necrotic core area. PAR-2 deficiency increased fibrous cap thickness and collagen content of plaque. Moreover, PAR-2 deficiency decreased smooth muscle cell content, macrophage accumulation, matrix metallopeptidase-9 expression and neovascularization in plaque. Relative quantitative PCR assay using thoracic aorta revealed that PAR-2 deficiency reduced mRNA expression of inflammatory molecules, such as vascular cell adhesion molecule-1, intercellular adhesion molecule-1, tumor necrosis factor (TNF)-α and monocyte chemoattractant protein (MCP)-1. *In vitro* experiment, we found that PAR-2 deficiency reduced mRNA expression of interferon-γ, interleukin-6, TNF-α and MCP-1 in macrophage under unstimulated and lipopolysaccharide-stimulated conditions. These results suggest that PAR-2 deficiency attenuates the progression and instability of atherosclerotic plaque.

## Introduction

Atherosclerosis is the result of a chronic inflammatory response in the arterial wall, which is initiated by the interaction of circulating monocytes with activated endothelial cells, followed by monocytes migration into the intima and subsequent uptake of low-density lipoprotein (LDL) by macrophages and their transformation into foam cells (Danzaki et al., 2012; De Paoli et al., 2014). Most of the morbidity and mortality associated with atherosclerosis occur due to acute coronary syndrome, which has a strong association with the formation and disruption of unstable atherosclerotic plaque (Libby and Theroux, 2005). The vulnerability and composition of plaque rather than the lesion size are the principal determinants of plaque stability and propensity to rupture (Fishbein, 2010). Sustained inflammation plays a vital role in the process of plaque formation and rupture. Accumulating evidence suggests that pro-inflammatory cytokines such as interferon (IFN)-γ, monocyte chemoattractant protein (MCP)-1 and interleukin (IL)-1 can promote the atherosclerotic plaque destabilization in apolipoprotein E-deficient (ApoE^-/-^) mice (Inoue et al., 2002; Koga et al., 2007; Alexander et al., 2012).

Protease-activated receptor (PAR)-2 is a G protein-coupled receptor that is activated by a unique mechanism of self-activation following specific proteolytic cleavage of their extracellular amino termini by a broad array of serine proteases and is known to mediate inflammatory processes in various tissues (Rothmeier and Ruf, 2012). Accumulating evidence suggests that PAR-2 activation promotes pro-inflammatory responses in many cell types, contributing to the pathogenesis of inflammatory diseases (Ragosta et al., 1994; Borensztajn et al., 2008; Tiwari et al., 2008; Rothmeier and Ruf, 2012). PAR-2 activity is known to be upregulated in the vasculature in inflammatory conditions (Hamilton et al., 2001). Inflammation increased PAR-2 expression in endothelial cells (Nystedt et al., 1996; Hezi-Yamit et al., 2005), in turn, PAR-2 activation promotes the production of pro-inflammatory cytokines such as tumor necrosis factor (TNF)-α, IFN-γ, IL-8 and IL-18 in the endothelium (Ikawa et al., 2005). We know that pro-inflammatory activation of endothelium recruits monocytes into inflamed vasculature, leading to the development of atherosclerosis. Moreover, PAR-2 expression is enhanced in human coronary atherosclerotic lesions (Napoli et al., 2004). The expression of PAR-2 is higher in atherosclerotic aorta in ApoE^-/-^ mice compared with aorta in wild-type mice (Hara et al., 2015). In our previous studies, we found that, PAR-2 activation promotes the expression of pro-inflammatory cytokines IL-6, IL-8, TNF-α and IFN-γ in macrophage, a key player in the progression and destabilization of atherosclerotic plaque (Zuo et al., 2015b). Thus, PAR-2 likely plays a role in the pathogenesis of atherosclerosis, but the potential relationship between PAR-2 and atherosclerosis is not clear. Therefore, in the present study, we aim to assess the effects of PAR-2 on the development and stability of atherosclerotic plaque by using mice that completely lack PAR-2 (PAR-2^-/-^) by crossing them with ApoE^-/-^ mice, which is the most common mice model for human atherosclerotic disease.

## Materials and Methods

### Animal and Induction of Atherosclerosis

The research was approved by the Local Animal Ethics Committee of Southeast University. The housing and care of animals and all the procedures done in the study were performed in accordance with the guidelines and regulations of the local Animal Ethics Committee of Southeast University. PAR-2^-/-^ female mice (Jackson Laboratory, Bar Harbor, ME, United States), at least 5 generations backcrossed to C57BL/6, were outcrossed to ApoE^-/-^ male mice (Jackson Laboratory, Bar Harbor, ME, United States), which had been backcrossed 11 generations to C57BL/6. PAR-2 wild-type and PAR-2^-/-^ mice among ApoE^-/-^ mice were designated as Control and PAR-2^-/-^, respectively. Male Control and PAR-2^-/-^ mice were fed a lard-containing diet comprising 21% lard and 0.15% added cholesterol for 12 weeks starting at 6 weeks of age. All mice were housed in the same room and exposed to the same light dark cycle.

### Animal Sacrifice and Preparation of Tissues

Mice were sacrificed at 18 weeks of age. Blood was collected from the heart into heparin containing tubes. Blood samples were centrifuged at 1000 *g* for 10 min at 4°C to obtain plasma, which was stored at -80°C until analysis. Then mice were perfused via the left ventricle with 5 ml PBS followed by 10 ml 4% paraformaldehyde. Thoracic aortas were dissected for subsequent gene expression analysis. Brachiocephalic arteries were dissected carefully, fixed overnight in 4% paraformaldehyde. Finally, brachiocephalic arteries were embedded in paraffin and serially sectioned (5 μm).

### Determination of Plasma Lipid Levels

Assays for determining plasma concentrations of total cholesterol, triglycerides, HDL and LDL cholesterol were performed by the Clinical Pathology Laboratory of Southeast University.

### Evaluation of Plaque Composition and Lesion Size

Elastic van gieson (EVG) staining was performed on slices for morphometric analysis of atherosclerotic lesions. Samples were analyzed by two independent blinded investigators and indices of atherosclerotic plaque were evaluated, including total lesion area, percentage of stenosis, thickness of fibrous cap and ratio of necrotic core. To detect collagen, Masson staining was performed on slices. These data were determined by using computer-assisted morphometry (Olympus, Tokyo, Japan) and used for subsequent statistical comparison.

### Immunohistochemistry

Tissue sections were dewaxed and rehydrated. Endogenous peroxidase activity was inhibited by incubation with peroxo-block (Invitrogen, Karlsruhe, Germany). After being blocked with 20% (vol/vol) goat serum in PBS, sections were incubated overnight at 4°C either with an anti-Mac-2 (Mac-2 antigen, a 32-kDa carbohydrate-binding protein expressed on the surface of inflammatory macrophages and several macrophage cell lines, which is a widely used surface marker of mouse macrophage development. Moreover, Thioglycolate-elicited macrophages express surface Mac-2 at higher levels than other macrophages. In our study, the macrophages used *in vitro* were obtained from thioglycolate-elicited peritoneal cavity, in order to ensure the consistency of *in vivo* and *in vitro* experiment, we chose Mac-2 to label the macrophages *in vivo* antibody (Santa Cruz Biotechnology, Santa Cruz, CA, United States), anti–α-SMA antibody (Santa Cruz Biotechnology, Santa Cruz, CA, United States), anti-CD 31 (which is regarded as evidence of neovascularization) antibody (Abcam, Cambridge, England) or anti-MMP-9 antibody (Santa Cruz Biotechnology, Santa Cruz, CA, United States) according to the manufacturers’ protocols. Sections then were incubated with the biotinylated secondary antibodies, rinsed 3 times with phosphate-buffered saline, and incubated for 10 min with streptavidin at room temperature. AEC-chromogen substrate (Invitrogen, Karlsruhe, Germany) was used for visualization. The extent of positive staining within the lesions was determined by using computer-assisted morphometry (Olympus, Tokyo, Japan). Specimens were scored according to the intensity of the dye color and the number of positive cells. The intensity of the dye color was graded as 0 (no color), 1 (light yellow), 2 (light brown), or 3 (brown); The number of positive cells was graded as 0 (<5%), 1 (5–25%), 2 (25–50%), 3 (51–75%), or 4 (>75%). The two grades were multiplied together and specimens were assigned to one of 4 levels: 0–1 score (-), 2 scores (+), 3–4 scores (++), more than 5 scores (+++) (Ma et al., 2010).

### Cell Culture

Macrophages were obtained as follows: 1 ml thioglycollate (4%) was intraperitoneally injected into mice and mice were sacrificed at the fourth day post injection. The belly skin was opened by pulling the skin apart at the middle of abdomen and abdominal membrane was not broken during the procedure. A 10 ml syringe attached to an 18G needle was used to inject 10 ml RPMI-1640 (with 10% FBS) into the abdominal cavity. The media were then slowly withdrawn back into the syringe and this was subsequently injected into 6 well plate (1.5–2 ml per well) and incubated at 37°C for 2 h. Supernatant was aspirated and then 2 to 3 ml fresh media was added before being incubated overnight. On the next day, the cells were harvested. Macrophages isolated from PAR-2^-/-^ and Control mice were cultured in RPMI-1640 supplemented with 100 μg/ml of penicillin, 100 μg/ml of streptomycin and 10% FBS. The cells were incubated in an atmosphere of 5% CO_2_ at 37°C and were routinely passaged every 3 days. To investigate the effects of PAR-2 on macrophage activation, we assessed the gene expression of TNF-α, IFN-γ, IL-6 and MCP-1 in unstimulated and LPS-stimulated macrophages that express or lack PAR-2.

### Relative Quantitative Polymerase Chain Reaction Assay

Total RNA was isolated from tissues and cells with TRIzol Reagent (Invitrogen, Carlsbad, CA, United States). cDNA synthesis was performed with the high-capacity cDNA archive kit (Applied Biosystems, Foster City, CA, United States). Relative quantitative PCR was performed to measure the mRNA levels of inflammatory cytokines (VCAM-1, ICAM-1, MCP-1, TNF-α, IFN-γ, IL-1β and IL-6) using a SYBR green PCR Kit (Roche, Basel, Switzerland).

### Statistical Analysis

Statistical analysis was performed by using SPSS 17.0. All data were represented as mean ± SD. Significant differences between means were determined with Student’s *t*-test or one-factor ANOVA. Ratio data were analyzed using Chi-square test. The statistical significance level was *P* < 0.05.

## Results

### PAR-2 Deficiency Attenuated Plaque Progression

**FIGURE 1:**
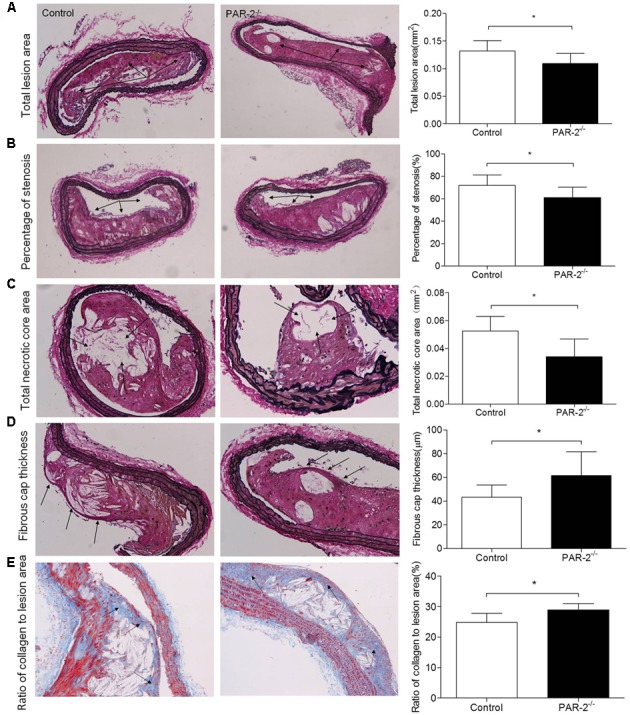
Morphometric analysis data. Representative images of EVG-stained brachiocephalic arteries exhibited significantly reduced total lesion area **(A)**, reduced percentage of stenosis **(B)**, reduced total necrotic core area **(C)** and increased fibrous cap thickness **(D)** in PAR-2^-/-^ mice. Representative images of Masson staining within brachiocephalic arteries showed significantly increased plaque collagen content **(E)** in PAR-2^-/-^ mice, as compared to the controls. Data represent mean ± SD. ^∗^*P* < 0.05.

During the entire experimental period, no mice died. Upon completion of lard-containing diet feeding, PAR-2^-/-^ mice did not significantly differ from control mice in body weight, plasma total cholesterol, triglyceride, high-density lipoprotein (HDL) and LDL cholesterol levels (**Table 1**). However, morphometric analysis of brachiocephalic arteries revealed that PAR-2 deficiency significantly attenuated progression of atherosclerotic plaques, there was a significant reduction in total lesion area (**Figure 1A**), percentage of stenosis (**Figure 1B**), and total necrotic core area (**Figure 1C**) between PAR-2^-/-^ mice and control mice.

**Table 1:** Body weight and lipids levels.

	Control (*n* = 8)	PAR-2^-/-^ (*n* = 8)	*P*
Body weight (g)	23.39 ± 4.15	23.61 ± 3.92	0.913
Total cholesterol (mmol/L)	15.56 ± 6.69	15.87 ± 6.11	0.924
Triglycerides (mmol/L)	3.01 ± 2.40	3.21 ± 2.41	0.873
LDL-cholesterol (mmol/L)	6.48 ± 2.34	6.14 ± 2.25	0.775
HDL-cholesterol (mmol/L)	3.57 ± 0.72	2.87 ± 0.77	0.079

*Differences were not significant between the study groups. Data represent mean ± SD. ^∗^*P* < 0.05*.

### PAR-2 Deficiency Promoted Stability of Atherosclerotic Lesion

**FIGURE 2:**
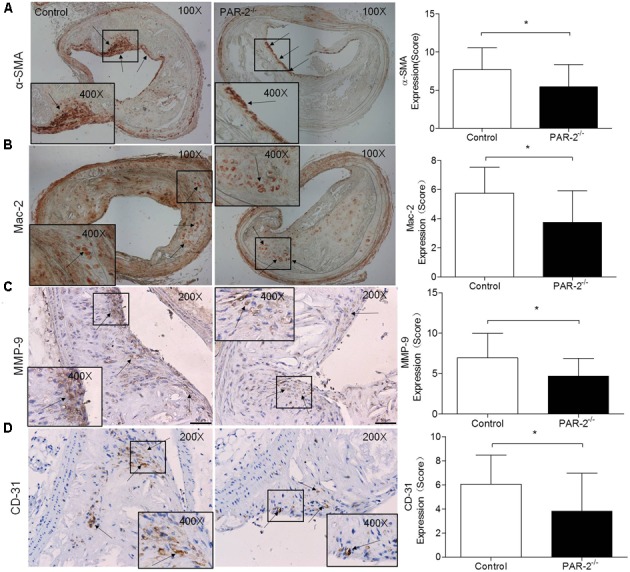
Immunohistochemistry. α-SMA **(A)** immunostaining for detecting plaque SMC content showed a significant reduction in PAR-2^-/-^ mice, as compared to the controls. Immunohistochemistry staining with antibody against Mac-2 **(B)**, which demonstrates the presence of macrophages within the atherosclerotic lesion, showed a significant reduction in PAR-2^-/-^ mice. Staining for MMP-9 **(C)** showed a significant reduction in PAR-2^-/-^ mice. Immunohistochemistry staining with antibody against CD-31 **(D)**, which is regarded as evidence of neovascularization, showed a significant reduction in PAR-2^-/-^ mice, as compared to the controls. **(C,D)** Magnified the shoulder regions of atherosclerotic plaques in the vessel. The arrows show positive stained areas within the atherosclerotic lesions. Data represent mean ± SD. ^∗^*P* < 0.05.

To determine whether PAR-2 deficiency alters stability of atherosclerotic lesion, plaques in brachiocephalic artery were analyzed for features which associated with plaque stability, such as fibrous cap thickness, collagen content, smooth muscle cell (SMC) content, macrophage content, and neovascularization (Fenning and Wilensky, 2014). First, PAR-2^-/-^ mice exhibited significantly increased protective fibrous cap thickness compared to the control mice (**Figure 1D**). Second, the ratio of collagen to total lesion area had a significant increase in PAR-2^-/-^ mice relative to control mice (**Figure 1E**). Third, the result of immunostaining against smooth muscle alpha actin (α-SMA) revealed that plaque SMC content was significantly decreased in PAR-2^-/-^ mice relative to controls (**Figure 2A**). Fourth, the result of immunostaining against Mac-2 revealed that PAR-2 deficiency significantly reduced accumulation of macrophage in atherosclerotic plaque compared to the control group (**Figure 2B**). Furthermore, immunostaining of matrix metallopeptidase (MMP)-9 showed a lower expression of MMP-9, which is responsible for the degradation of fibrillar collagen, in plaques of PAR-2^-/-^ mice compared to control mice (**Figure 2C**). Finally, immunohistochemistry confirmed that PAR-2 deficiency resulted in a significantly reduced neovascularization, as measured by staining against CD-31, compared with control mice (**Figure 2D**). We also evaluated intraplaque hemorrhage, which is associated with and contribute to plaque instability (Kolodgie et al., 2003; Takaya et al., 2005). However, intraplaque hemorrhage was not observed in both groups. Thin fibrous cap, collagen loss, increased macrophage accumulation and neovascularization in plaque are the features of unstable plaque (Alexander et al., 2012). Taken together, these data indicated that PAR-2 deficiency promoted stability of atherosclerotic lesion.

### PAR-2 Deficiency Reduced Vascular Inflammation

**FIGURE 3:**
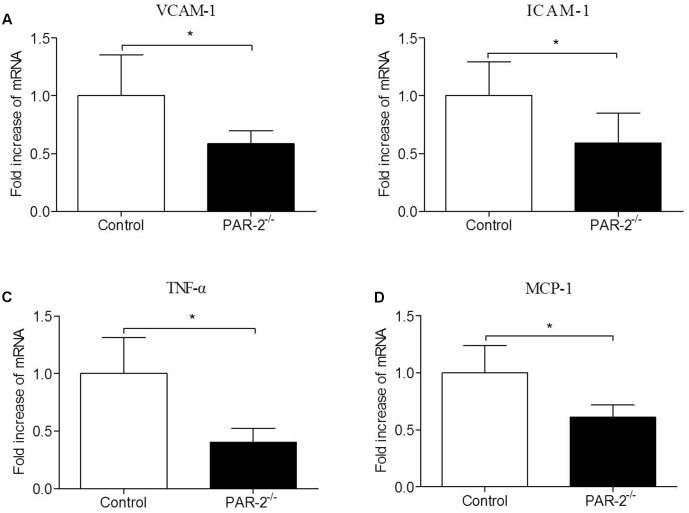
Relative quantitative PCR assay for vascular inflammation. PAR-2 deficiency significantly decreased mRNA expression of VCAM-1 **(A)**, ICAM-1 **(B)**, TNF-α **(C)** and MCP-1 **(D)** in aortic tissues of PAR-2^-/-^ mice, as compared to the controls. Data represent relative fold of mRNA expression as compared to the control group. Data represent mean ± SD. ^∗^*P* < 0.05.

To investigate whether PAR-2 deficiency affected vascular inflammation, we assessed inflammatory gene expression in thoracic aorta using PCR. PAR-2 deficiency reduced mRNA expression of inflammatory mediators, such as vascular cell adhesion molecule (VCAM)-1, intercellular adhesion molecule (ICAM)-1, TNF-α and MCP-1 (**Figure 3**). It is consistent with the result of immunohistochemistry, the expression of macrophage marker Mac-2 in PAR-2^-/-^ mice was reduced significantly.

### PAR-2 Enhanced Macrophage Inflammatory Responsiveness

**FIGURE 4:**
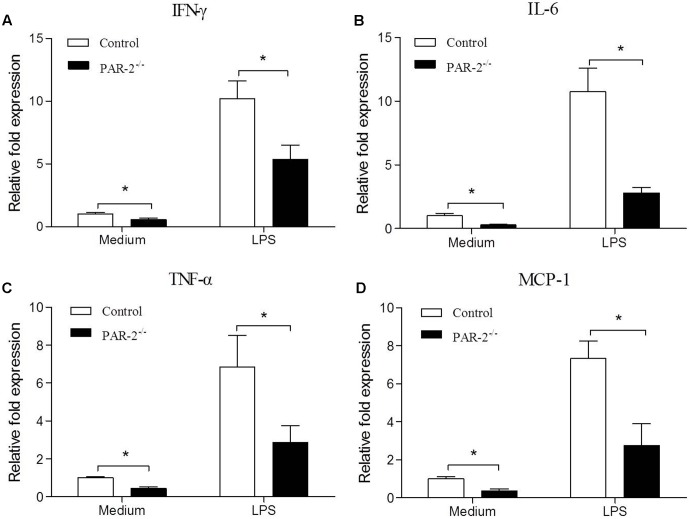
Relative quantitative PCR assay for macrophage inflammatory responsiveness. Relative quantitative PCR analysis revealed that unstimulated and LPS-stimulated PAR-2^-/-^ macrophage produced significantly lower levels of IFN-γ **(A)**, IL-6 **(B)**, TNF-α **(C)** and MCP-1 **(D)** compared with the control macrophage. Data represent relative fold of mRNA expression as compared to the control group. Data represent mean ± SD. ^∗^*P* < 0.05.

To investigate the molecular mechanism by which PAR-2 affected the development and stability of atherosclerotic lesion, we assessed the gene expression of IFN-γ, IL-6, TNF-α and MCP-1 in unstimulated and LPS-stimulated macrophages that express or lack PAR-2. PCR analysis revealed that PAR-2^-/-^ macrophages expressed significantly lower levels of IFN-γ, IL-6, TNF-α and MCP-1 compared with the control macrophages under unstimulated and lipopolysaccharide (LPS)-stimulated conditions (**Figure 4**). These findings suggest that PAR-2 enhances the inflammatory responsiveness in macrophages, promoting the expression of major inflammatory mediators which are involved in the development of atherosclerosis.

### PAR-2 Deficiency Inhibited Macrophage Adhesion to Smooth Muscle Cell

**FIGURE 5:**
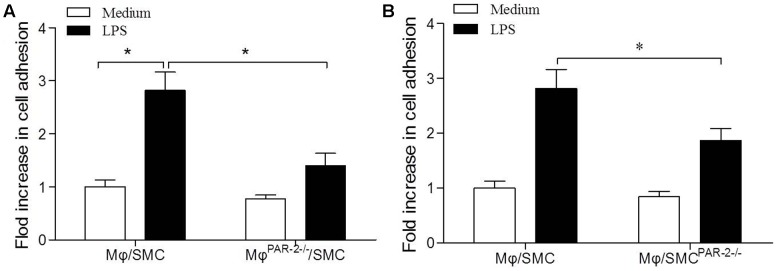
Cell adhesion assay. Mφ was co-cultured with SMC for 15 min in presence (black bars) or absence (white bars) of LPS. LPS induced a significant increase in Mφ adhesion to SMC by approximately threefold compared to unstimulated cells **(A)**. Mφ from PAR-2-deficient mice (Mφ^PAR-2-/-^) significantly reduced LPS-induced adhesion **(A)**. SMC from PAR-2-deficient mice (SMC^PAR-2-/-^) induced a significant inhibitory effect on LPS-induced adhesion **(B)**. Results are presented as fold change of adhesion relative to medium treatment. Data represent mean ± SD. ^∗^*P* < 0.05.

To assess macrophage (Mφ) adhesion to SMC, we used a co-culture model consisting of a 15 min incubation of SMC and Mφ. In order to produce a pro-inflammatory environment, we incubated both cell types separately with LPS before the co-culture incubation. We found that LPS induced a significant increase in Mφ adhesion to SMC by approximately threefold compared to unstimulated cells (**Figure 5A**). To further explore the role of PAR-2 in cell interactions, we then co-cultured Mφ from PAR-2-deficient mice (Mφ^PAR-2-/-^) with SMC from control mice, we found that PAR-2 deficiency significantly reduced LPS-induced adhesion (**Figure 5A**). Similarly, when Mφ from control mice were co-cultured with SMC from PAR-2-deficient mice (SMC^PAR-2-/-^), PAR-2 deficiency induced a significant inhibitory effect on LPS-induced adhesion (**Figure 5B**). The results demonstrate that the pro-adhesive action of PAR-2 occurs via effects on both Mφ and SMC.

### PAR-2 Induced a Bidirectional Paracrine Communication between Mφ and SMC

**FIGURE 6:**
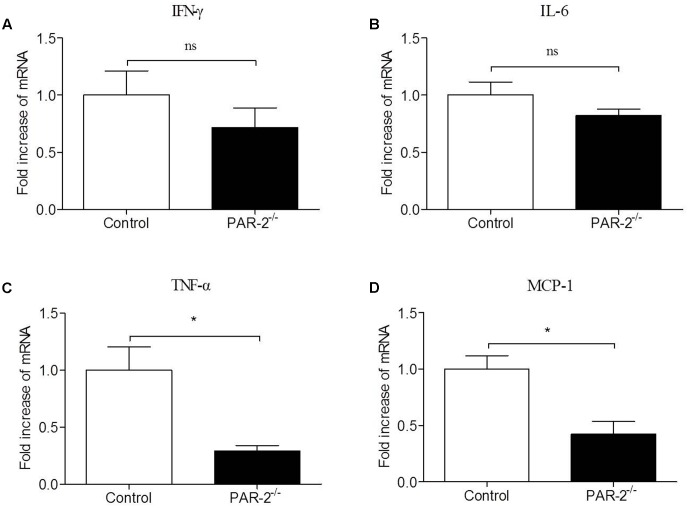
Culture medium from SMC^PAR-2-/-^ reduced inflammatory responsiveness in Mφ. SMC from control mice and PAR-2-deficient mice (SMC^PAR-2-/-^) were preincubated with LPS, subsequently cell-free culture supernatants from SMC or SMC^PAR-2-/-^ were transferred to untreated Mφ cultures. Relative quantitative PCR analysis revealed that culture medium from SMC^PAR-2-/-^ did not affect the expression of IFN-γ **(A)** and IL-6 **(B)** in Mϕ, induced a significant down-regulation of TNF-α **(C)** and MCP-1 **(D)** expression in Mφ. Data represent relative fold of mRNA expression as compared to the control group. Data represent mean ± SD. ^∗^*P* < 0.05.

**FIGURE 7:**
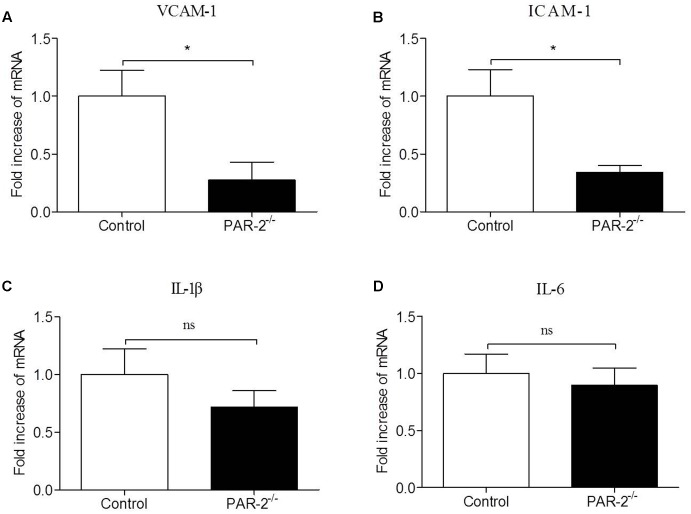
Culture medium from Mφ^PAR-2-/-^ reduced inflammatory responsiveness in SMC. Mφ from control mice and PAR-2-deficient mice (Mφ^PAR-2-/-^) were preincubated with LPS, subsequently cell-free culture supernatants from Mφ or Mφ^PAR-2-/-^ were transferred to untreated SMC cultures. Relative quantitative PCR analysis revealed that culture medium from Mφ^PAR-2-/-^ induced a significant down-regulation of VCAM-1 **(A)** and ICAM-1 **(B)** expression in SMC, did not affect the expression of IL-1β **(C)** and IL-6 **(D)** in SMC. Data represent relative fold of mRNA expression as compared to the control group. Data represent mean ± SD. ^∗^*P* < 0.05.

To verify whether a paracrine PAR-2-triggered cross talk of Mφ and SMC could possibly occur, we transferred conditioned cell-free culture supernatants from SMC^PAR-2-/-^ to Mφ cultures or vice versa, and analyzed the cells for the expression of inflammatory genes. We found that culture supernatants from SMC^PAR-2-/-^ induced a significant down-regulation of TNF-α and MCP-1 expression in Mφ (**Figure 6**). In turn, when culture supernatants from Mφ^PAR-2-/-^ were transferred to SMC, VCAM-1 and ICAM-1 expression in SMC were decreased significantly (**Figure 7**). The results above provide experimental evidence that PAR-2 is capable to induce a bidirectional paracrine pro-inflammatory reaction between Mφ and SMC.

## Discussion

Atherosclerosis is a chronic inflammatory process of vessel walls and characterized by recruitment of circulating inflammatory cells, such as monocytes and activated T-cells (Galkina and Ley, 2009; Danzaki et al., 2012). Accumulating evidence suggests that PAR-2 occupies a crucial position in inflammation and regulates vascular function (Al-Ani et al., 1995; Steinhoff et al., 2005), but little is known about the proatherosclerotic effects of PAR-2. In this study, PAR-2 deficiency attenuated the progression of atherosclerotic lesions in ApoE^-/-^ mice. In addition, PAR-2 deficiency promoted multiple features of atherosclerotic plaque stability, including increased plaque collagen content, reduced macrophage accumulation and reduced neovascularization. *In vitro* experiment, we found that PAR-2 contributes to pro-inflammatory activation of macrophage. These results suggest that PAR-2 deficiency attenuates the progression and instability of atherosclerotic plaque.

The plaque collagen content, which plays an important structural role in stabilizing plaques and is decreased in unstable lesions (Burleigh et al., 1992; Crisby et al., 2001; Neumeister et al., 2002; Verhoeven et al., 2005), determines the integrity and strength of protective fibrous cap. In addition to increased protective fibrous cap thickness, PAR-2 deficiency promoted features of plaque stability, such as increased plaque collagen content in this study. The new collagen within plaques is mainly produced by SMCs, meanwhile, collagen degradation is due to the expression of active MMPs. Evidence suggests that SMCs play an important role in enhancing atherosclerotic plaques stability (Schwartz et al., 2000; Clarke et al., 2006). The expression level of MMPs is associated with stability of plaques, at least in part through their ability to degrade the collagen (Newby, 2006). Accumulating evidence suggests that collagen degradation is more important than collagen synthesis in determining the integrity and strength of fibrous cap (Galis, 2004; Ray et al., 2004). High expression of MMPs is closely related to increased collagen degradation and decreased fibrous cap thickness (Steen and O’Donoghue, 2013; Zuo et al., 2015a). It is consistent with the results in our study, the marked reduction in MMP-9 and α-SMA levels within the plaques of PAR-2-deficient mice, combined with the increased plaque collagen content, suggest that the thickening of the fibrous cap may be mainly due to the lower expression of MMP-9.

Inflammation is recognized as a prominent feature of atherosclerosis, inflammatory events such as monocyte recruitment, macrophage activation and cytokine production are critical for atherosclerotic lesion progression and destabilization (Roselaar et al., 1996; Zhou et al., 2000; Song et al., 2001; Hansson and Hermansson, 2011). Macrophage recruitment and activation are dependent on the expression of endothelial adhesion molecules, monocyte chemoattractants and pro-inflammatory cytokines (Boring et al., 1998; Galkina and Ley, 2007). In the present study, we found that PAR-2 deficiency strongly attenuated atherosclerotic plaque progression and destabilization and that this atheroprotective effect was associated with reduced macrophage accumulation in the atherosclerotic plaque and decreased expression of VCAM-1, ICAM-1, TNF-α and MCP-1 in the thoracic aorta. To strengthen our theory, we examined the effect of PAR-2 on the inflammatory response of macrophage *in vitro*, we found that macrophage isolated from PAR-2^-/-^ mice express significantly lower levels of TNF-α, IFN-γ, IL-6 and MCP-1 than macrophage isolated from the controls. Although these data cannot establish cause and effect, they strongly suggest that PAR-2 deficiency attenuates atherosclerotic plaque progression and destabilization, at least in part, by suppressing vascular inflammation and pro-inflammatory activation of macrophage.

We know that cell adhesion is typically stimulated by cytokines such as TNF-α released from inflammatory cells and vascular SMCs (Warner and Libby, 1989). The adhesion of macrophages to vascular SMCs can be enhanced through the actions of TNF-α (Wirrig et al., 2016). TNF-α leads to an up-regulation of VCAM-1 and ICAM-1, which facilitate macrophage adhesion (Galkina and Ley, 2007). Combined with the results in our study, we infer that PAR-2-induced bidirectional paracrine pro-inflammatory reaction between Mφ and SMC may contribute to the pro-adhesive action of PAR-2.

The neovascularization within the atherosclerotic plaque is a key element in the plaque stability. PAR-2 signaling is sufficient for the pro-angiogenic effect (Uusitalo-Jarvinen et al., 2007). PAR-2 deficiency interferes with normal revascularization and the activation of PAR-2 can enhance revascularization (Sitaras et al., 2015). It is consistent with the result of our study, in the present study, CD-31, which is regarded as the evidence of neovascularization, was reduced markedly in atherosclerotic lesions of PAR-2^-/-^ mice. The result suggests that the inhibition of neovascularization in plaque may provide a possible mechanism for the plaque-stabilizing effects of PAR-2 deficiency.

**FIGURE 8:**
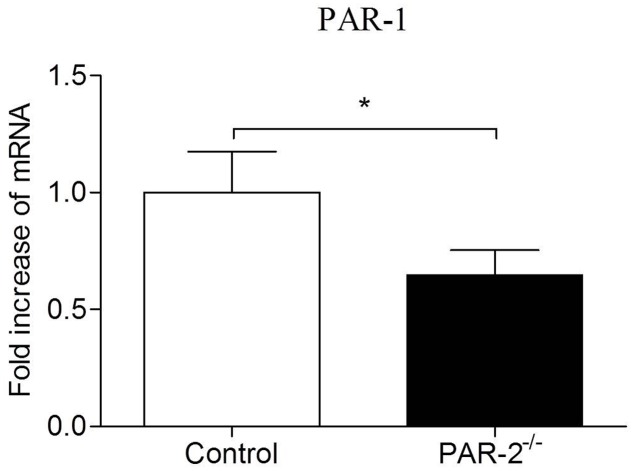
Relative quantitative PCR assay for PAR-1. Relative quantitative PCR analysis revealed that there was a significantly reduced expression of PAR-1 in aortic tissues of PAR-2^-/-^ mice, as compared to the controls. Data represent relative fold of mRNA expression as compared to the control group. Data represent mean ± SD. ^∗^*P* < 0.05.

In recent years, compelling evidence suggests that PAR-1 and PAR-2 may facilitate each other’s activity in different pathophysiological processes (Lin et al., 2013). PAR-1 and PAR-2 can directly interact pharmacologically and act as a functional unit forming heterodimers that induce different signaling pathways compared to those induced by monomers (Kaneider et al., 2007; Lin and Trejo, 2013). Atherosclerosis is a chronic inflammatory process of vessel wall, inflammatory condition can promote the formation of PAR-1/2 complexes, PAR-2 plays a dominant role in the process (Nystedt et al., 1996; Kaneider et al., 2007). McEachron et al. (2010) found that mammary adenocarcinoma cells lacking PAR-2 failed to express PAR-1 in response to thrombin activation. It’s consistent with our findings in the present study that the expression of PAR-1 on aortas from PAR-2 deficient mice was decreased (**Figure 8**). During vascular inflammation, PAR-1 and PAR-2 can signal as an entwined pair, highlighting a complex, and PAR-2 appears to dominate over PAR-1 in the cooperative signaling between PAR-1 and PAR-2 (Mirza et al., 1996; Nystedt et al., 1996; Kaneider et al., 2007). Furthermore, PAR-2 forms a stable complex with PAR-1 and modulates PAR-1-driven SMC de-differentiation and proliferation in response to vascular injury (Sevigny et al., 2011). In consideration of the results that PAR-2 deficiency reduced vascular inflammation and SMC levels in our study, combined with the decreased expression of PAR-1 in PAR-2 deficient mice, we speculate that the lack of interaction between PAR-1 and PAR-2 is, at least in part, responsible for the enhanced stability of atherosclerotic plaque.

Apolipoprotein E is an exchangeable apolipoprotein which plays a critical role in regulating plasma cholesterol levels. Emerging roles beyond lipoprotein metabolism are being recognized increasingly for ApoE in the pathogenesis and treatment of inflammatory diseases on the basis of its ability to suppress inflammation (White et al., 2014). The anti-inflammatory effects of ApoE are independent of its cholesterol-lowering property (Gaudreault et al., 2012). Increasing ApoE expression in monocytes can induce an anti-inflammatory phenotype and reduce the recruitment of inflammatory cells to sites of injury (Baitsch et al., 2011; Potteaux et al., 2011). Transduction of ApoE in macrophages of atherosclerotic ApoE-deficient mice significantly reduces lesion formation without affecting plasma cholesterol levels (Curtiss and Boisvert, 2000). Moreover, accumulating evidence suggests that feeding a high-fat diet to ApoE-deficient mice can cause pro-inflammatory response (Yao et al., 2016). In our study, we used ApoE-deficient mice rather than wild-type mice to establish the animal model of atherosclerotic lesion, therefore it is not clear whether the anti-inflammatory response linked to PAR-2 deficiency is in relation to the absence of ApoE. It is the limitation of our study, the potential interaction of ApoE and PAR-2 in inflammation should be studied in the further research.

Taken together, results of the present study provide evidence that PAR-2 deficiency plays a protective role in the development and stability of atherosclerotic plaque.

## Author Contributions

PZ and GM conceived and designed the experiments. PZ, ZZ, and YZ performed the experiments. PZ and XW analyzed the data. QZ and LC contributed reagents/materials/analysis tools. PZ wrote the paper. All authors discussed the results and commented on the manuscript.

## Conflict of Interest Statement

The authors declare that the research was conducted in the absence of any commercial or financial relationships that could be construed as a potential conflict of interest.
